# The developmental transcriptome dynamics of current-year shoot utilized as scion in *Camellia chekiangoleosa*

**DOI:** 10.1186/s12870-025-06715-3

**Published:** 2025-05-28

**Authors:** Yu Sheng, Haili Gao, Chunlian Yu, Guangyuan Huang, Kunxi Wang, Kailiang Wang, Leyan Lv, Wei Long

**Affiliations:** 1 State Key Laboratory of Tree Genetics and Breeding, Key Laboratory of Tree Breeding of Zhejiang Province, Research Institute of Subtropical Forestry, Chinese Academy of Forestry, Hangzhou, 311400 China; 2 Zhejiang Public Welfare Forest and State Forest Farm Management Station, Hangzhou, 310019 Zhejiang China; 3 Quzhou Doctoral Innovation Workstation, Changshan Country Oil Tea Industry Development Center, Quzhou, 323900 China; 4 College of Hydraulic Engineering, Zhejiang Tongji Vocational College of Science and Technology, Hangzhou, 311231 Zhejiang China

**Keywords:** Transcriptome analysis, Shoot development, *Camellia chekiangoleosa*

## Abstract

**Background:**

*Camellia chekiangoleosa* is the most widely planted red-flowered and large-fruited oil-*camellia* species, with high value in edible oil production and landscaping. To better understand the weak scion development and slow graft-union healing underlying grafting propagation challenges in *C. chekiangoleosa*, we conducted temporal RNA-seq on current-year shoots with five time points determined according to changes in cell wall composition, aiming to reveal dynamic developmental regulation.

**Results:**

Analysis of temporal expression characteristics of genome-wide genes and differentially expressed genes (DEGs) revealed that genes differentially patterned between stem and apical bud were enriched in functions related to cell division and differentiation, hormone responses, and vascular or flower development. Coexpression network analysis revealed that red/far-red light and gibberellin (GA) signaling were closely correlated with flowering development in *C. chekiangoleosa* shoots. We further analyzed a unique module showing a negative correlation between the module and traits (cell wall composition, i.e., lignin, cellulose, and hemicellulose content). Genes in the top-scored sub-cluster of this module were enriched in shoot development-related processes, including cell wall dynamics, xylem development, secondary cell wall biogenesis, lignin biosynthesis, and procambium histogenesis. WOX4 and PXY, known markers of cambium cells, were identified as key hub genes, along with the actin-binding protein WLIM1. These coexpressed hub genes associated with vascular system development peaked in June in stems and were validated by qRT-PCR, suggesting that June may be an optimal grafting season for *C. chekiangoleosa*.

**Conclusions:**

Integrating transcriptomics and physiology defined the dynamic signature of changes in composition of cell wall and gene activity during the development of current-year shoots in *C. chekiangoleosa*. Our findings provide insights into a potential molecular strategy for breeders, targeting key regulators specific to cambium differentiation, and physiological strategy for hormone or light supplied artificially to enhance grafting productivity of *C. chekiangoleosa*.

**Supplementary Information:**

The online version contains supplementary material available at 10.1186/s12870-025-06715-3.

## Introduction

Oil-*Camellia*, the general term for dozens of *Camellia* genus (Theaceae family) tree species valued for edible oil production, was historically explored directly from natural populations and is now extensively cultivated for its seed oils'nutritional and health benefits. In China, *oil-camellia* plantation productivity is projected to reach 6 million hectares by 2025, with an estimated gross production value of 55 billion USD annually [1]. *Camellia chekiangoleosa* (*C. chekiangoleosa*) is an endemic variety of oil-*Camellia* in China, has a shorter reproductive cycle (6 months, flowering in March and fruit ripening in September). This species is increasingly prioritized due to its large fruits, high seed oil content, and ornamental red flowers, making it valuable for oil producing, breeding, and landscaping [2–4]. Nurse-seedling grafting remains the primary method for asexual mass propagation of high-quality seedlings required for large-scale oil-camellia cultivation [5]. However, the growing demand for elite grafted seedlings faces challenges: slow graft-union healing and a scion supply–demand imbalance caused by weak transverse diameter growth and insufficient stem node numbers in current-year shoots—key traits related to grafting productivity.

Both empirical practices and biological research have established that scion quality critically influences the growth potential of grafted seedlings [6, 7]. Grafting biology, encompassing molecular mechanisms of scion-rootstock compatibility, interactions, and traits improvement (e.g., stress tolerance and fruit quality and yield) [8], has become a focal area in contemporary botanical research. In oil-*Camellia*, scions-rootstocks interactions has been observed to affect root growth in grafted seedlings [9]. A recent metabolomic study of scion buds across oil-*Camellia* varieties revealed variety-specific metabolite profiles, with correlation analysis identifying metabolites linking to grafting success and growth capacity [5]. Additionally, the lignification degree of scions significantly impacts grafted plant survival and growth in oil-*camellia* [10, 11]. Thus, understanding the characteristics and growth mechanism of shoots, the precursor of scion explants, is critical for the efficient cultivation and management of the scions, and the subsequent growth of grafted seedlings.

Extensive studies on morphogenetic, physiological and genetic aspects of stem cell fate decision in shoot apical meristems (SAM), bud dormancy/broken, and stem development, and endogenous-exogenous regulatory networks have been conducted in economically significant species, including crop and fruit species, or woody trees [12–16]. Transcriptomics has advanced understanding of shoot growth mechanisms in model systems such as *Arabidopsis thaliana* and *Populus* spp.. Recent spatial transcriptome analyses in *Populus* uncovered dynamic cambium differentiation trajectories during primary and secondary growth based on tissue-specific marker genes [17, 18].

Despite these advances, shoot development of *C. chekiangoleosa* remains poorly understood**,** overshadowed by research on its key economic traits (e.g., yield and oil content). Here, integrated dynamic transcriptional profiling and cell wall composition analysis of *C. chekiangoleosa* shoot to identify key regulators of apex-to-stem developmental transitions, aiming to enhance grafting productivity through science-based management strategies for commercial cutting orchards.

## Methods

### Sample collection

This study used 12-year-old clonally propagated healthy *C. chekiangoleosa* plants grown in an open-field germplasm nursery in Changshan County, Zhejiang Province (28°86′365′′ N, 118°45′55′′ E). Current-year growing branches were collected, and apical buds and stems were manually dissected, immediately frozen in liquid nitrogen, and store at −80 ℃, respectively. Three biological replicates were sampled at five developmental stages: May 14, May 21, June 05, June 21, and July 01.

### Determination of lignin, cellulose, and hemicellulose content in shoots

All experiments performed in triplicate with 40–60 mesh milled tissues from stems and apical buds. Samples were extracted with benzene-ethanol (2:1, v/v) using a Soxhlet for 4 h, and air-dried completely for cell wall composition analysis.

Total lignin (acid-insoluble lignin **[**AIL**]**) + acid-soluble lignin **[**ASL**]**) was quantified via a two-step acid hydrolysis method [19]. AIL: Air-dried samples (1.0 g, W_1_) were hydrolyzed with 15 mL 72% H_2_SO_4_ (v/v) and agitated continuously at 30 °C for 3 h. The mixture was diluted with ultrapure water, boiled for 4 h, and vacuum-filtered through a G2 crucible. Residues were dried at 105 °C to constant weight (W₂), ashed at 560 °C for 2 h (W₃), and calculated as AIL% = (W_2_–W_3_)/W_1_ × 100%. ASL: Filtrates were diluted to 250 mL with 2.88% H₂SO₄. Absorbance at 205 nm (UV spectrophotometer) was used to calculate ASL% = A × D × V/1000 × K × W_1_ × 100%, where A = absorption value, D = dilution ratio, K: absorptivity = 110 L/g/cm.

#### Cellulose

Air-dried samples (0.3 g) was hydrolyzed with 3 mL 72% H_2_SO_4_ (v/v) at 30 °C for 1 h, then diluted with 84 mL ultrapure water, autoclaved (121 ℃, 1 h) and filtered. Glucose content was detected using a D799407 Glucose content assay kit (Sangon Biotech Company, Shanghai, China), and converted to cellulose content (glucose content × 0.90) [20].

#### Hemicellulose

Calculated as holocellulose (%) − cellulose (%), following the Chinese National Standard GB/2677.10–1995.

### Transcriptome sequencing, assembly and functional annotation

Apical bud and stem samples with four and five stages respectively (one bud sample was accidentally damaged) were used for library construction and sequencing.

RNA was isolated using TRIzol reagent (Invitrogen), quantified with a Bioanalyzer 2100 and RNA 6000 Nano LabChip Kit (Agilent), and sequenced on an Illumina Hiseq-4000 (LC-Bio, Hangzhou, China). Filtered read pairs with adapter and low-quality bases trimmed using Cutadapt and Perl script, were quality-checked using FastQC (http://www.bioinformatics.babraham.ac.uk/projects/fastqc/). High-quality clean reads were used for de novo assembly with Trinity 2.4.0 program [21]. The longest transcripts of each cluster unit were selected as unigenes. Salmon [22] and edgeR [23] were respectively used for transcript abundance estimation and pairwise differential expression analysis with the threshold of |log2 FC|≥ 1 and false discovery rate (FDR) < 0.05. Unigenes were annotated against databases including NCBI non-redundant (NR), Gene Ontology (GO), Kyoto Encyclopedia of Genes and Genomes (KEGG), SwissProt, Pfam, and eggNOG database (E-values < 1e-5). The output of Illumina sequencing, quality controlling, and gene annotation was detailed in Suppl. Table S1 ~ S3 and Suppl. Fig. S1. ClusterPofiler 4.4.4 R package was used for GO enrichment analysis and visualization [24].

### Gene clustering and coexpression network analysis

The expression pattern of genes and DEGs was analyzed using clusterGVis (version 0.1.1) [25] and Mfuzz (version 2.58.0) [26] package, respectively. A weight gene coexpression network (WGCNA) was conducted using the WGCNA Shiny plugin in TBtools toolkit [27]. The default parameters were used for noise removal, except for the expression cutoff and the second filter method were set as 2 and Var, respectively. Soft-thresholding power = 12, min module size = 40, and module cuttree height = 0.25. Cytoscape 3.9.1 was employed for networks visualization.

### qRT-PCR validation

The PrimeScript™ 1 st RT Master Mix (Perect Real Time) Kit (TaKaRa, Dalian, China) was used for reverse transcription from each RNA sample according to the manufacturers’ instructions. The primer pairs were listed in Suppl. Table S4. The qRT-PCR programs were performed on the ABI PRISM 7300 real-time PCR system (Applied Biosystems, USA) using TB Green Premix Ex Taq™ II (Tli RNaseH Plus) (TaKaRa, Dalian, China), and programmed referring to a previous study [28]. Data were analyzed using the 2^−ΔΔCT^ method with three biological replicates and using a ‘GAPDH-q’ gene in *C. oleifera* as the reference gene.

### Statistical analysis

Data are presented as mean ± SD (three replicates). Differences between time points were assessed by one-way ANOVA with Duncan’s post hoc test (*p* < 0.05).

## Results

### Analysis of lignin, cellulose, and hemicellulose content in *C. chekiangoleosa* shoot development

**Fig. 1:**
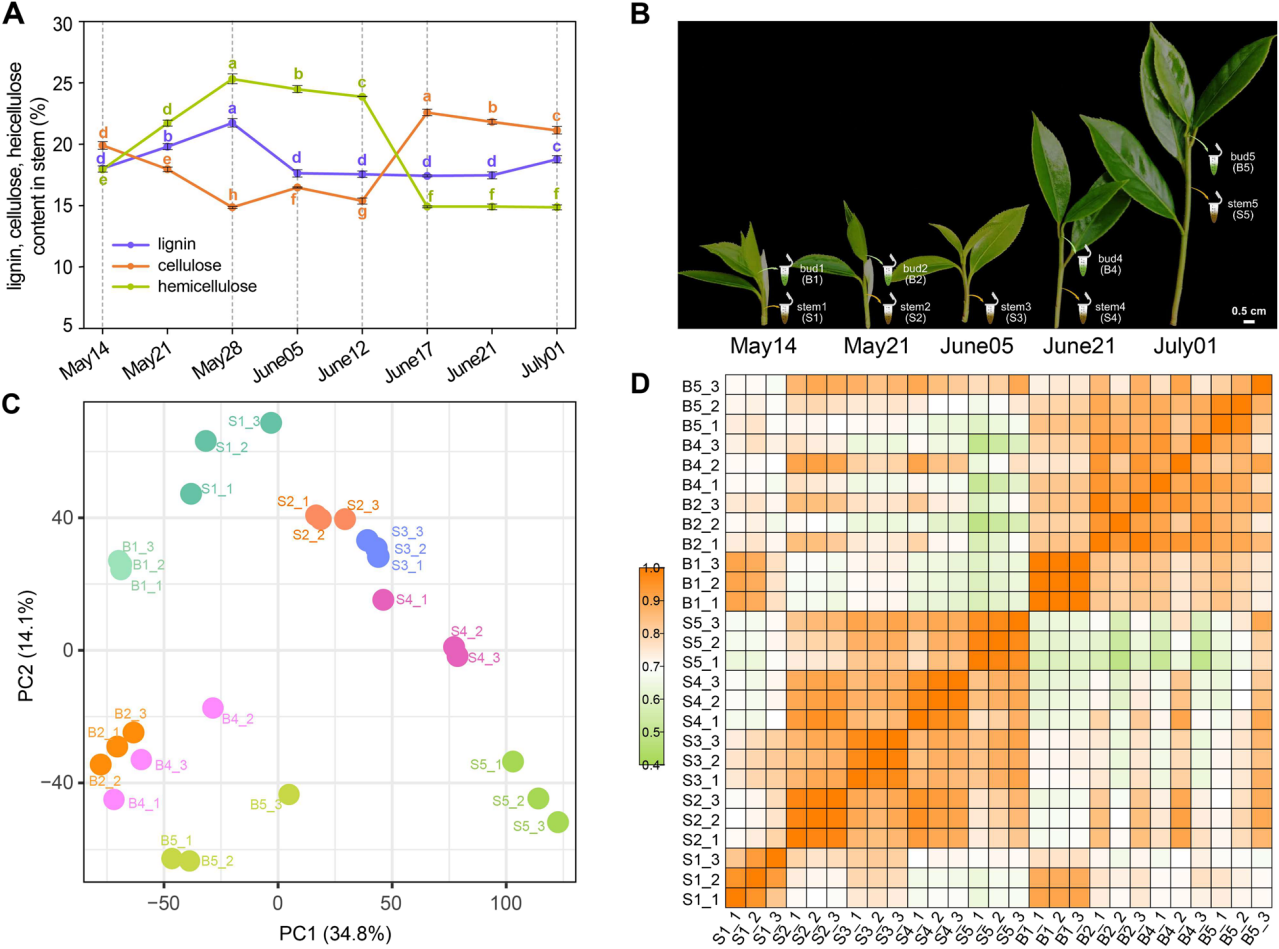
Composition changes of cell wall in shoot and assessment of RNA-seq. **A** Content detection of lignin, cellulose, and hemicellulose in stem of developing* C. chekiangoleosa* shoot were presented as weight percentage of the dry mass (w/w %). Error bars represent the mean ± SD (n = 3). Different letters indicated significant differences (P < 0.05) according one-way ANOVA followed by a Duncan’s test. **B** Stem and bud samples collected for RNA-sequencing. Sampled apical bud and stem tissues were orderly named after tissue type and the exact sampling dates (14 May, 21 May, 05 Jun, 21 Jun, 01 Jul). **C** PCA plot showed a gene expression difference between tissue types of stem and bud. Expression data of genes were log10 (TPM + 1)-transformed before the PCA was implemented using the rgl package. **D** Transcript abundances were highly correlated between replicate samples

To investigate lignification progression during shoot development in *C. chekiangoleosa*, we monitored cell wall composition changes in current-year-shoot continuously from May to July, the major grafting season for oil-*Camellia* species (Fig. 1A, Suppl. Fig. S2). Developing apical buds exhibited lower and relatively stable lignin content across all stages examined. In both stem and apical bud, hemicellulose constituted a higher proportion before June 12. In contrast to apical bud, the contents of lignin, cellulose, and hemicellulose in stem were initially more similar on 14 May, then tended to be divergent, and diverged most by 28 May, following an opposite change for cellulose and hemicellulose levels between 12 June ~ 17 June, with a relative plateau before and after that. However, the lignin content was strongly declined at 28 May, then progressed essentially unchanged in the relatively long time. Primary cell walls typically exhibit a low cellulose-to-hemicellulose ratio. We observed a pronounced increase in cellulose content on 12 June, suggesting a critical shift for developmental trajectories of primary growth and secondary growth in stem. This cellulose accumulation likely enhances mechanical strength, preparing shoots for subsequent fruit expansion in July.

### Overview of global expression patterns and function classification of genes during *C. chekiangoleosa* shoot development

To investigate developmental dynamics of *C. chekiangoleosa* scions at the transcriptional level, 27 independent libraries representing two tissues (stem and bud, with 5 and 4 stages, respectively) were constructed and sequenced (Fig. 1B). Principal component analysis (PCA) of all the expressed unigenes (TPM ≥ 1) showed clear separation between stem and bud samples (Fig. 1C), suggesting tissue-specific transcriptional profiles. High correlation between biological replicates (Pearson’s correlation coefficients: 0.82–0.89) confirmed data reproducibility (Fig. 1D).

To profile genome-wide transcriptome changes during *C.chekiangoleosa* shoot development, 44,418 unigenes with an average TPM ≥ 1 in at least one phase were k-means clustered into 9 groups (G1 ~ G9) (Suppl. Fig. S3). Genes in both G1 and G2 were significantly enriched in functions related to photosynthesis, and response to light stimulus, were strongly highly expressed at the first bud stage in G1 and G2, and at the S1 stage in G1 (Suppl. Fig. S3). G3 consisted of genes that were enriched in hormone response, cell division and differentiation, and were strongly downregulated at the last stage of stem and bud (S5 and B5) (Suppl. Fig. S3, blue triangle). Genes assigned to G8 and G9 were noted as being enriched in functions related to shoot development and displayed distinct expression trends across different stages between the two tissues, stem and bud (Suppl. Fig. S3). In G8, genes were up-regulated gradually and possessed the strongest expression at the S2 stage in stem tissues, then decreased in fluctuation, and were enriched in GO terms of secondary cell wall biogenesis, xylem and phloem pattern formation, xylem development, cell wall organization or biogenesis, and biosynthesis of glucuronoxylan, the major hemicellulose component of plant secondary walls (Suppl. Fig. S3, red triangle). Similar function classification such as phloem development and secondary cell wall biogenesis were also over-represented by genes in G9 (Suppl. Fig. S3, red star). Expression of these genes showed progressive uptrend, but dramatically increased in stem at the final B5 stage (Suppl. Fig. S3). By contrast, genes clustered into G8 and G9 transcriptionally changed gently among different stages during bud development (Suppl. Fig. S3), reflecting DEGs associated with stem development-related functions played tissue-specific roles.

### Identification of DEGs during *C. chekiangoleosa* shoot development

**Fig. 2:**
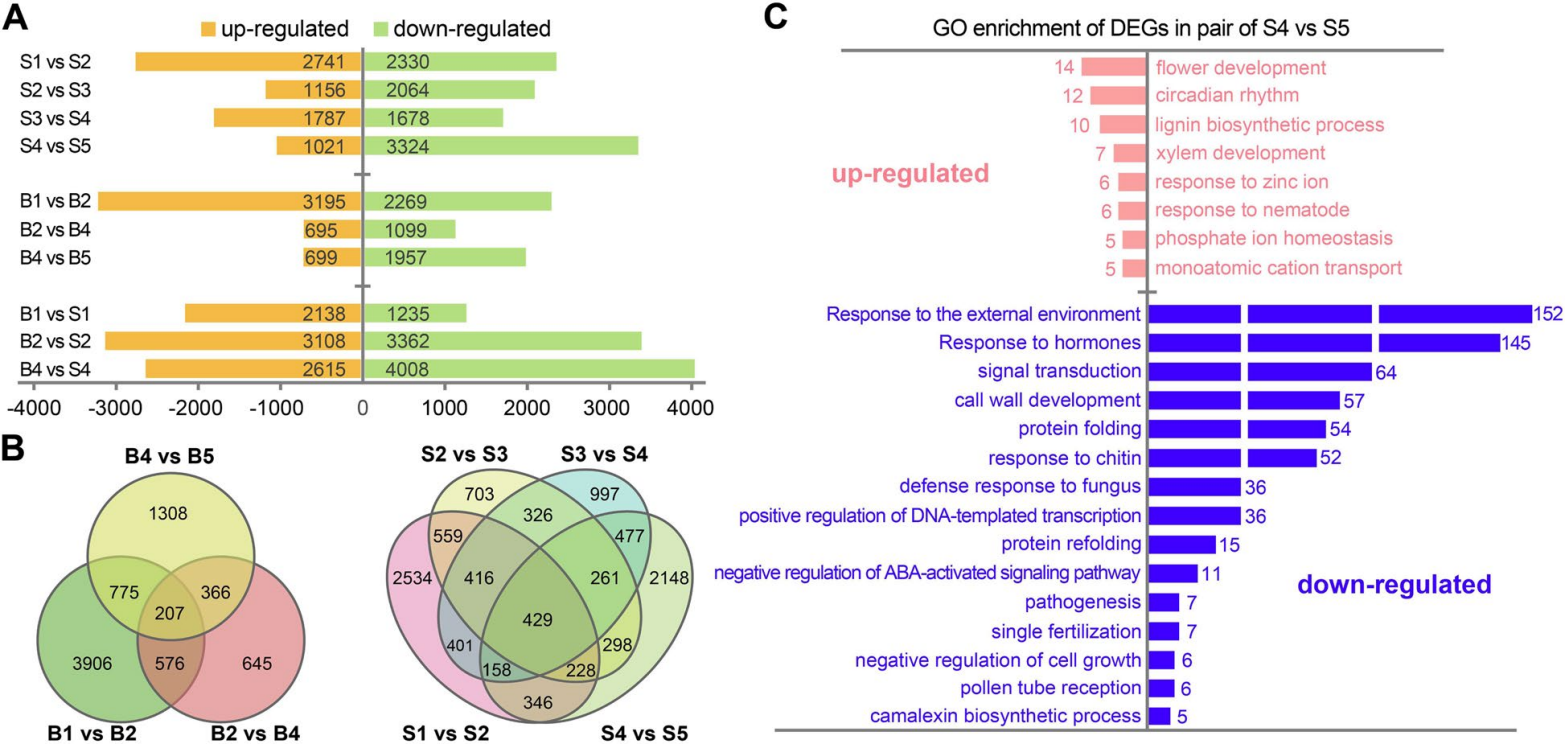
Gene differentially expressed between adjacent or the same developmental stage. **A** Barplot showing the number of DEGs identified from different pairwise-comparation groups. **B** Venn diagram showing the shared and unique DEGs in tissues of stem or apical bud compared to adjacent stages. **C** Representative GO terms enriched by up- and down-regulated DEGs in S4 vs S5 pairwise-comparation. Numbers beside the columns indicate gene count of each term

To explore the regulatory factors behind the morphological and functional heterogeneity at different stages during *C. chekiangoleosa* shoot development, we analyzed genes differentially expressed by pairwise comparisons between adjacent stages, and between the different tissues at the same time points. Based on the following filters: fold change ≥ 2.0, FDR < 0.05, more DEGs were found in pairwise comparisons of the first two stage, that is, S1 vs S2 in stem, and B1 vs B2 in apical bud (Fig. 2A), suggested greater developmental changes occurred in shoot during this period. Additionally, smaller number of DEGs were found when comparing S1 stem and B1 apical bud (Fig. 2A), suggesting a comparatively minor degree of heterogeneous development between the stem and the apical bud at the initial stage.

Venn diagrams showed that there were 206 and 420 DEGs shared among pairwise comparisons between adjacent stages within apical bud and stem tissue, respectively (Fig. 2B). These findings suggest that gene expression changed at different developmental stages in different tissues. Notably, stem tissues at different stages shared more DEGs (Fig. 2B), which are likely to have significant functional importance in controlling *C. chekiangoleosa* shoot development. Curiously, a marked imbalance was observed between the number of DEGs up-regulated and down-regulated in the comparison of S4 vs S5, with the latter is three times greater the former (Fig. 2A). Functional enrichment was carried out to explore the biological significance underlying such imbalance. Up-regulated DEGs were more involved in functions related to flower development, circadian rhythm, lignin biosynthesis, and xylem development, in contrast to that down-regulated DEGs were more represented in functions of response to external environment and hormones, and cell wall development (Fig. 2C, Suppl. Table S5).

### Divergence in expression pattern of DEGs across developmental stages between stem and bud tissues

**Fig. 3:**
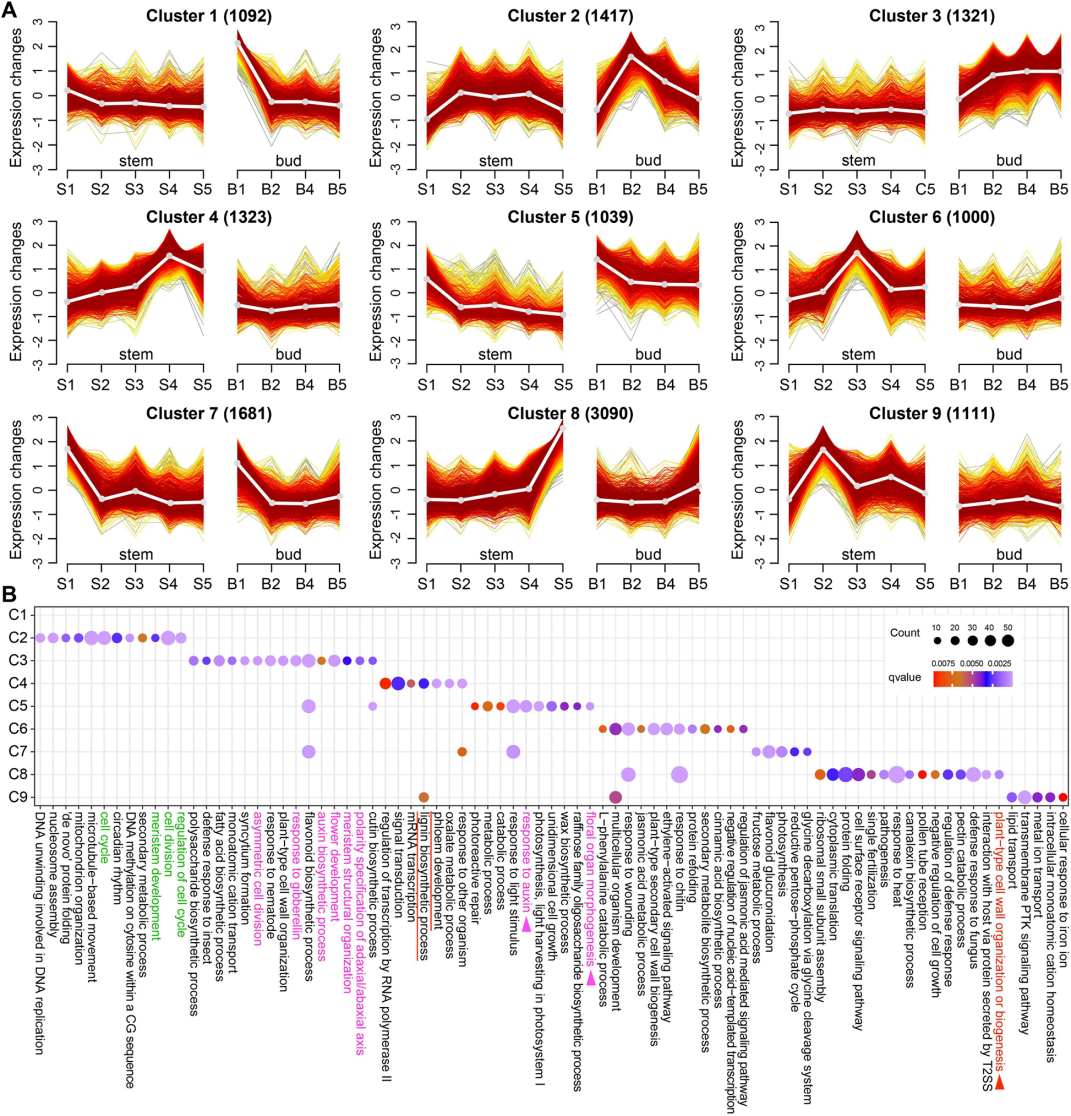
Temporal expression pattern of DEGs between stem and apical bud. **A** Gene clusters identified in 27 samples. A line for the cluster center were shown in white. **B** Top 15 biological process categories of each cluster. GO terms with q-value < 0.01 were submitted to revigo for simplification using default semantic similarity measure and a personal setting with 0.5

To further define the temporal characteristics and functional categories of the DEGs as a whole, we performed fuzzy c-means clustering analysis of 13,075 genes that were expressed differently across adjacent stages of stem and apical bud. This analysis grouped all the DEGs into 9 clusters (C1 ~ C9, Fig. 3A).

In general, the expression levels of C1, C5, C7 genes rapidly decayed from the 1 st stage (S1, B1) to the 2nd stage (S2, B2), and then slightly changed to the 5 th stage (S5, B5) (Fig. 3A). Function related to light response was overrepresented by genes in both C1 and C7 (Suppl. Table S6). Additionally, numerous G7 genes related to glucosyltransferase or glycosyltransferases were enriched in the term of flavonoid biosynthetic process. The peak expression of genes in C2, which functioned in cell cycle or cell division and meristem development, was observed in apical bud stage B2 (Fig. 3A, and Fig. 3B in green font). C8, comprising the largest number of genes, accounted for ~ 23.6% of the total genes examined (Fig. 3A). C8 genes are mainly expressed at the last stage S5 in stem and are involved in biological processes such as stress (heat, wounding, and plant pathogens) response and regulation, as well as cell wall organization or biogenesis (Fig. 3A and 3B). The cell wall-related functional category were mainly enriched by 5 annotated as cellulose synthase-like protein coding genes (Fig. 3A and 3B; Suppl. Table S6), which are still largely functionally unknown. In *Arabidopsis*, the cellulose synthase-like (AtCSL) genes have been classified into six subfamilies, AtCSLA ~ AtCSLG. The AtCSLD3 was previously reported to be a Golgi-localized beta-glycan synthase that polymerize the backbones of hemicelluloses of cell wall and is related to root hair elongation [29]. Genes in C3 and C5 were observed to have higher expression in the 2nd to 5 th stage of apical bud than in stem. GO enrichment analysis showed that C3 and C5 genes functioned in categories of hormone responsiveness and meristem development and differentiation, such as response to GA, auxin biosynthetic process, flower development, meristem structural organization, polarity specification of adaxial/abaxial axis (pink font), or floral organ morphogenesis, response to auxin (pink font and arrow) (Fig. 3B). This suggested a possible role for GA and auxin applied to C. chekiangoleosa shoot apex during phase change and meristem development, consistent with numerous studies that indicate crosstalk between auxin and GA signaling during reproductive transition and the regulation of shoot morphogenesis in plants [30, 31]. C4 genes, which are involved in lignin biosynthesis and phloem development, tended to have enhanced expression in stem from S1-S4 (Fig. 3A, and Fig. 3B underlined in red). These results revealed possible signature events in a certain phase, driven by differentially functionalized genes that were respectively highly expressed in the two tissues during the developmental transition from apical bud to stem.

We further explored the tissue-specific identity genes associated with cambium differentiation in each cluster. TRINITY_DN18953_c0_g8 and TRINITY_DN21805_c0_g5, annotated as cambium cell marker genes *WUSCHEL-related homeobox 4* (*WOX4*) and *TDIF RECEPTOR* (*TDR*)*/PHLOEM INTERCALATED WITH XYLEM* (*PXY*), respectively, were assigned to C4 or C6 and reach their expression peaks at S3 and/or S4 stage (Suppl. Table S7). Two genes potentially encoding the meristem identity genes *AINTEGUMENTA* (*ANT*) (TRINITY_DN14771_c1_g4, TRINITY_DN15537_c2_g5, and TRINITY_DN22858_c0_g7) and one gene encoding *SHORT ROOT* (*SHR*) (TRINITY_DN12209_c0_g1), were mapped to C4 or C6 and were highly expressed at stage S3 or S4 (Suppl. Table S7). In addition, four predicted phloem identity genes in C7, including *BETA-AMYLASE 3* (*BAM3*) (TRINITY_DN14816_c1_g2), *DNA binding with one finger* (*Dof3.6*) (TRINITY_DN13829_c0_g3 and TRINITY_DN13829_c0_g1), and *SMAX1-LIKE 3* (*SMXL3*) (TRINITY_DN14770_c1_g10), exhibited the typical expression trends observed in C7 (Fig. 3, Suppl. Table S7). Besides, all predicted sieve or vessel marker genes *SIEVE ELEMENT OCCLUSION C* (*SEOC*), *SIEVE ELEMENT-OCCLUSION-RELATED 1* (*SEOR1*), *ENDO-BETA-MANNASE 6* (*MAN6*) peaked at S4 or S5 in the stem, suggesting the most active period of secondary cell wall formation.

### Light and hormone-associated factors involved in apical bud development in *C. chekiangoleosa* shoot

**Fig. 4:**
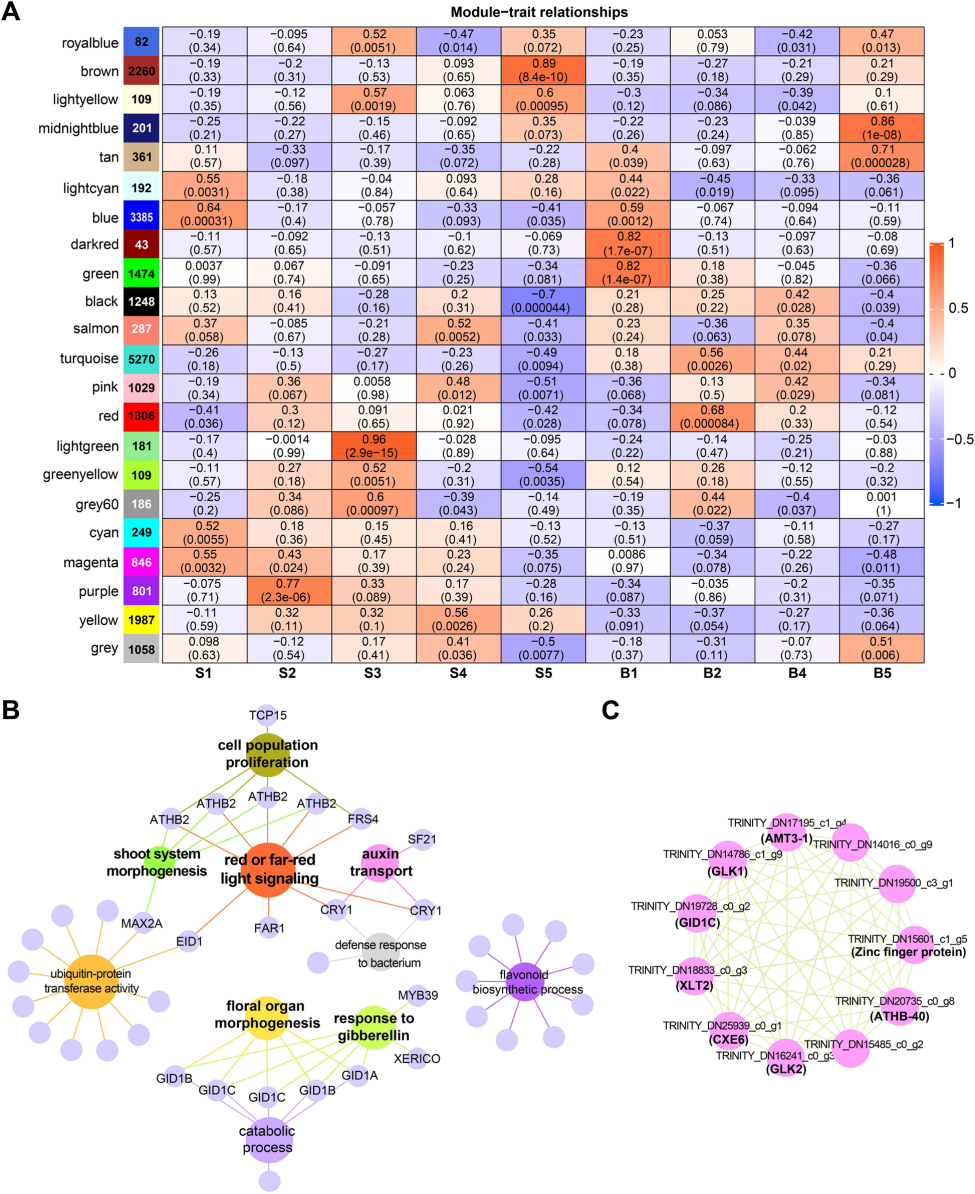
Co-expression modules that were highly specific to developmental stage. **A** Module– tissue association matrix. The dark red or dark blue color of each cell at the row–column intersection indicates a high degree of positive and negative correlation between a module and the tissue, respectively. The numbers in bold font in the left color blocks showed the size of genes assigned to a specific module. **B** GO functional categories of genes in the tan module. **C** Correlation network of hub genes in B5-related tan module. The hub genes were selected according to the standard with kME > 0.80 and GS > 0.80

WGCNA is effective in detecting phenotypes-specific gene sets or functional pathways. Herein, we built co-expression networks to identify clusters of highly interconnected genes that were highly related to the developmental stage of stem or apical bud in *C. chekiangoleosa* current-year-shoot. For noise removing, all genes that with a TPM less than 2 in more than 90% of the samples were eliminated, followed by a second filter based on default Var method. 22,946 retained genes were ultimately classified into 22 distinct co-expression modules, containing genes with numbers ranging from 82 (royalblue) to 5270 (turquoise) (Fig. 4A, Suppl. Fig. S4). We observed seven modules, including purple, lightgreen, brown, darkred, green, midnightblue, and tan, which were highly positively correlated with stage S2, S3, S5, B1, or B5 respectively, with the criteria of r^2^ > 0.7 and *p* < 0.001 (Fig. 4A).

In the B5-related tan module, GO terms of red or far-red light signaling, shoot system morphogenesis, and/or cell population proliferation were enriched by multiple genes annotated as *ARABIDOPSIS THALIANA HOMEOBOX PROTEIN 2* (*ATHB-2*), and *FAR1-RELATED SEQUENCE like* (*FRS4*), among others (Fig. 4B). Besides, one phytochrome-related gene *EMPFINDLICHER IM DUNKELROTEN LICHT 1* (*EID1*), two cryptochrome-related genes *Cryptochrome-1* (*CRY1*), and one *FAR-RED IMPAIRED RESPONSE 1* (*FRS4*) were classified under the functional categories of auxin transport and/or far-red light signaling (Fig. 4B). Two GO terms, GA response and floral organ morphogenesis, were mainly enriched by multiple GA receptors including *INSENSITIVE DWARF1 A* (*GID1 A*), *GID1B*, and *GID1 C* (Fig. 4B), suggesting the association of components of the GA signaling pathway during apical bud differentiation and flowering in *C. chekiangoleosa* shoot development. Furthermore, GA receptor *GID1 C*, as well as one *CARBOXYLESTERASE 6* (*CXE6*), which belongs to the same *CXE* family as *GID1s* [32], were also identified as two of the 11 hub genes in the apical bud B5 stage-related tan module (Fig. 4C). This further supports the potential role of GA-related factors in the development of *C. chekiangoleosa* apical buds.

### Coexpression network analysis reveals key factors in *C. chekiangoleosa* stem development

**Fig. 5:**
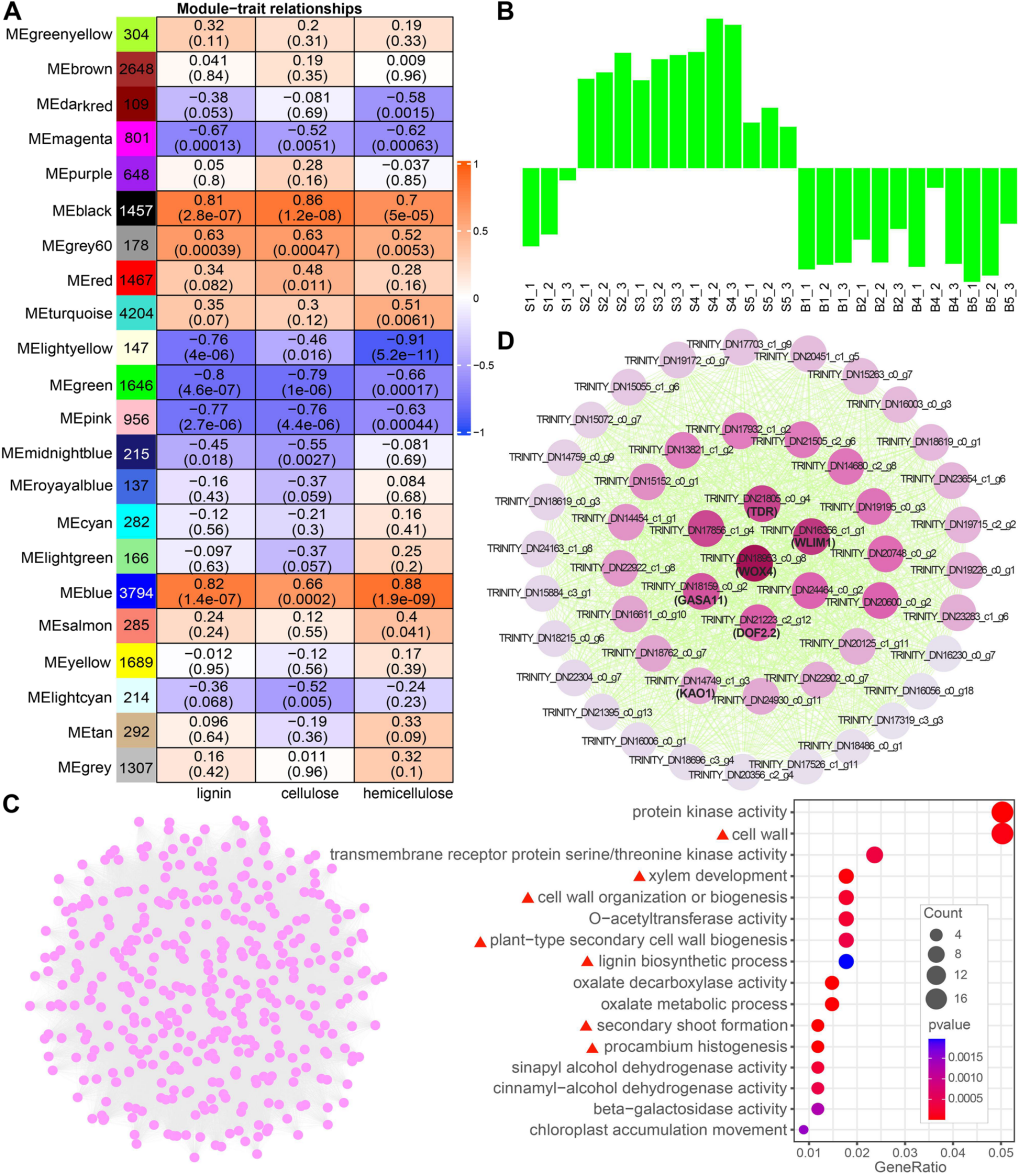
Analysis of gene co-expression and association between genes and wall composition. **A** Module-cell wall components association. The color of each cell at the row–column intersection indicates the correlation coefficient between the module and the content of lignin, cellulose, and hemicellulose. The values in each cell indicate the correlation coefficient and *p*-value. **B** Eigengene expression profile showing the TPM of genes in the green modules. **C** Network visualization and functional enrichment analysis of genes in the top highest ranked predicted cluster screened from the network of green module. **D** Subnetwork with the top 50 genes generated from the top MCODE cluster in (**C**). Light–dark gradient ranked by scores range from low to high

When analyzing the co-expression modules that were highly associated with certain stages, we additionally noted the yellow module possessed a higher positive correlation with the stem than with the bud (Fig. 4A). Genes in this yellow module (Fig. 4A) were similar to those in the C4 cluster from the clustering analysis of the expression profile (Fig. 3A), as well as functional subcategories involving secondary cell wall development, such as lignin biosynthesis, xylem or phloem development (Fig. 3B and Suppl. Fig. S5, underlined in red). Therefore, we further investigated the associations between co-expressed modules and the three most abundant structural carbohydrates (lignin, cellulose, and hemicellulose) in the stem of *C. chekiangoleosa* using WGCNA (Fig. 5).

Among the 22 modules identified, five modules (black, blue, lightyellow, green, and pink), were found to be highly positively or negatively correlated with at least one of these wall components (Fig. 5A). The green module was negatively correlated with lignin, cellulose, and hemicellulose. The expression profile showed that genes in the green module were upregulated, except for their low expression in stem at the S1 stage (May 04) and also downregulated in bud over the entire period (Fig. 5B).

A top-ranked central gene cluster comprising 399 genes was further screened from this green module using the Molecular Complex Detection (MCODE) plugin in Cytoscape V3.9.1 (Fig. 5C, Supplementary Fig. S6). GO enrichment analysis showed that genes in the top MCODE cluster were enriched in function related to protein kinase activity, as well as plant shoot development-relative processes including cell wall, xylem development, secondary cell wall biogenesis, lignin biosynthetic process, and procambium histogenesis (Fig. 5C, red triangle).

Subsequently, the top 50 hub genes of this top MCODE cluster were predicted using the Cytohubba plugin (Fig. 5D). In which, four genes TRINITY_DN16356_c1_g1 (annotated as LIM domain-containing protein *WLIM1*), TRINITY_DN20125_c1_g11, TRINITY_DN17703_c1_g9, and TRINITY_DN15884_c3_g1 were annotated as actin- relative factors (Fig. 5D, Suppl. Table S8). Besides, two genes TRINITY_DN17856_c1_g4 and TRINITY_DN14749_c1_g3 were annotated as Gibberellin-regulated protein 11 (*GASA11*) and *ENT-KAURENOIC ACID OXIDASE 1* (*KAO1*), respectively, with *KAO1* being a key regulator of GA biosynthesis (Fig. 5D, Suppl. Table S8). The other top-ranked scored hub genes were TRINITY_DN18953_c0_g8 (annotated as *WOX4*), TRINITY_DN21805_c0_g4 (annotated as *TDR*/*PXY*), and TRINITY_DN21223_c2_g12 (annotated as *DOF2.2*) (Fig. 5D, Suppl. Table S8). In particular, TRINITY_DN18953_c0_g8 (*WOX4*), scored the highest among the hub genes (Fig. 5D, Suppl. Table S8), which acted as a central regulator in cambial activity. The coexpression of these genes associated with plant vascular tissue development suggests an important role of green module in stem growth of *C. chekiangoleosa*.

## Discussion

### Coordinated light and GA signaling cues for flowering development in the shoot apex of *C. chekiangoleosa*

Plant shoot ontogeny differentiates into juvenile and adult stages [33]. The juvenile phase starts with the proper specification of the shoot meristem to initiate stems, leaves, buds, internodes, and axillary buds. In *C. chekiangoleosa*, new shoot growth persists for approximately three months until transitioning to flowering in late June [34].

Genes associated with cell proliferation and red/far-red light signaling were enriched by genes (*ATHB-2*, *TCP15*) of B5 (apical bud sampled on July 01)-related tan coexpressed module (Fig. 4B). *ATHB-2* and *TCP15* are reported as negative regulators of specific cell proliferation processes [35, 36]. The former is tightly induced by far-red-rich light signal causing morphological changes [37], the latter bind to and activate positive flowering regulating gene *SOC1* (*SUPPRESSOR OF OVEREXPRESSION OF CONSTANS1*) [38]. One speculation is that red/far-red light signaling appears to mediate vegetative growth arrest in the stem apex to stop elongating continually, facilitating reproductive initiation.

Consistent with the onset of morphological differentiation in late June in *C. chekiangoleosa* [34], WGCNA revealed that the B5-related tan module was enriched for flower organ development and GA response functions, mainly via GA receptor genes (Fig. 4B). Among these, *GID1C* and *CXE6* (a member of the CXE family closely related to *GID1*) were identified as hub genes,**,** both peaked in expression in B5 stage (Suppl. Fig. S7). GA signaling plays essential roles in controlling flowering development of plants [39]. GID1s are key components of the Gibberellin-GID1-DELLA pathway [40] and *GID1 s* triple mutants exhibit delayed flowering and defective floral organ development [39]. These results imply synergistic roles for GA and red/far-red light signaling in coordinating flowering in *C. chekiangoleosa* shoot.

Previous studies report that exogenous GA application influence flower bud differentiation and shoot development in oil-*Camellia* species [41, 42]. Spraying optimal concentrations of GA before the physiological differentiation period of flower buds significantly promotes flower bud formation and internode spacing of spring shoots in both *Camellia oleifera* and *Camellia osmantha*, as well as the shoot diameter of the latter [42]. This inspires that GA supplied exogenously to serve as an agricultural tool to control current-year-shoot development in *C. chekiangoleosa* to enhance grafting productivity.

### *WOX4 *is suggested essentially functioned in *C. chekiangoleosa* shoot development

Plants exhibit continuous apical and lateral growth through the activity of vascular stem cells, with the procambium and cambium serving as critical meristematic tissues responsible for generating xylem and phloem [43], which collectively support structural integrity and deliver resource, such as water, nutrients, photosynthetic products and signaling molecules, needed for growth and defense [43]. *WOX4* is regarded as the central regulator of cambial activity since it integrates multiple pathways, including hormonal and *TDIF*/*PXY* signaling [44]. Besides being downstream of *TDIF*/*PXY* signaling, *WOX4* is required for *ARF5*-mediated repression of cambium activity and ethylene-mediated promotion of cambium activity [44]. In *Populus tomentosa*, auxin response factor *PtoARF7* directly activates *WOX4*, showing a synergetic regulation on cambial activity with GA [45].

In *C. chekiangoleosa*, cambium cell marker (*WOX4*, *PXY*) and meristem identity genes (*ANT*, *ATHB8*, *SHR*) showed a steep decrease of expression from initial to S2, fluctuating at high levels and finally decreasing. Sieve and vascular marker genes (*SEOR1*, *XCP2*, and *MAN6*) showed a general increase in expression before peaking at S4 or S5 (Suppl. Table S7). This expression pattern supported that the dynamic balance between primary and secondary growth in current-year-shoot of *C. chekiangoleosa* is orchestrated by transcriptional network involved in cambium activity.

The identification of WOX4, *PXY*, *Dof2.2* and GA signaling factor *GASA1* as top hub genes in the coexpression green module, which is negatively correlated with lignin, cellulose, and hemicellulose content (Fig. 5, Suppl. Table S8), highlights their dual role in both suppressing secondary cell wall deposition and maintaining cambium proliferative activity. *WOX4* and *PXY* were regarded as cambium cell markers [17], and some members of the Dof family were marker genes related to phloem identity [18]. This suggested a similar regulatory network, centered on *WOX4* for maintenance of cambium activity and secondary growth in shoot developmental control, likely existed in *C. chekiangoleosa*.

### WOX4 seems to be indicating the best timing of graft in* C. chekiangoleosa*

Shoot is the structure of the plant mainly including stem, which firmly supports leaves, and buds that timely develop into flowers and fruits on ontogeny [46]. When used as scions, shoots rely on secondary vascular tissue development for post-grafting growth. Lignification, which confers mechanical strength to shoot tissues, is essential for the load-bearing capacity of current year shoots that need to support heavy fruits in *C. chekiangoleosa*. For *C. chekiangoleosa*, mainly propagated by nurse seedling grafting in actual production, lignification degree of the scion is determined as an important index contributing to grafting success [5, 11].

Cambium and procambium are considered as key tissues that enable tissue attachment and vascular differentiation during successful grafting [47]. Development of the secondary vascular tissue of the shoots using as scions affects a lot of growth of the whole grafted plant. Therefore, genes involved in the relatively well-characterized process of cambium development, e.g., junction formation, are potential targets for improving *C. chekiangoleosa* shoot.

*WOX4* is a central regulator maintaining cambial activity, which plays crucial roles from primary to secondary growth [48]. The expression of *WOX4* negatively correlated with lignin, cellulose, and hemicellulose content during stem development of the current-year-shoots in *C. chekiangoleosa* (Fig. 5A). Transcriptional profiling of *WOX4* was more likely to marker period where there was strong activity of cambial development in *C. chekiangoleosa* current year shoot. Recent studies highlight WOX4’s crucial role in vascular reconnection during grafting, suggesting its utility as an early indicator of graft failure [49, 50]. In our results, *WOX4* and coexpressed vascular development genes peaked in June in *C. chekiangoleosa* stem (Fig. 5B), confirmed by qRT-PCR (Suppl. Fig. S8). We asked if June would be a more suitable time when the graft success is a seasonal-related trait? This remains to be concluded from specific data on grafting practices in *C. chekiangoleosa*. Notably, commercial grafting of *C. chekiangoleosa*, is predominantly conducted in June, aligning with this finding. Altogether, genetically modulating *WOX4* expression may serve as a viable strategy for breeders to influence cambium differentiation and enhance grafting success. Further exploration of WOX4’s role may advance vegetative propagation techniques for this species.

## Conclusions

Using transcriptomics and biochemical assays, we provide the first dynamic transcriptional profile and the compositional changes (lignin, cellulose, and hemicellulose content) of the current-year shoot development in *C. chekiangoleosa*. The data indicate that genes related to cell division and differentiation, hormone responses, and vascular or flower development promote the differential development between stem and apical bud. Gene module linking to in GA and red/far-red light signaling was identified to be substantially associated with flowering development, supporting the strategies for artificial regulation of *C. chekiangoleosa* shoot development. We proposed a possible regulatory network, centred on *WOX4*, for coordinating cambium activity and secondary growth in the control of *C. chekiangoleosa* shoot development. Peak expression existing in June of the key hub genes (*WOX4*, *PXY*, *WLIM1*) of this network inspires that June may be an optimal grafting season for *C. chekiangoleosa*. These key regulators specific to cambium differentiation, specifically *WOX4*, deserve further investigation to help improve grafting productivity in *C. chekiangoleosa* in the future.

## Supplementary Information

Supplementary Material 1

Supplementary Material 2

Supplementary Material 3

Supplementary Material 4

Supplementary Material 5

Supplementary Material 6

Supplementary Material 7

Supplementary Material 8

Supplementary Material 9

Supplementary Material 10

Supplementary Material 11

Supplementary Material 12

Supplementary Material 13

Supplementary Material 14

Supplementary Material 15

Supplementary Material 16

## Data Availability

Raw reads in this study have been submitted to NCBI under bioproject (PRJNA1103664).

## Abbreviations

*DEGs*: Differentially expressed genes
*GA*: Gibberellin
*SAM*: Shoot apical meristems
*AIL*: Acid-insoluble lignin
*ASL*: Acid-soluble lignin
*WGCNA*: Weight gene coexpression network
*PCA*: Principal component analysis
*MCODE*: Molecular Complex Detection
