# Supplementary material for: The developmental transcriptome dynamics of current-year shoot utilized as scion in Camellia chekiangoleosa
Source: BMC Plant Biol. 2025 May 28;25:712. doi: 10.1186/s12870-025-06715-3 (PMC12117948; doi:10.1186/s12870-025-06715-3)
Supplement: Supplementary file 1 — Supplementary Material 1 [file 12870_2025_6715_MOESM1_ESM.pdf]

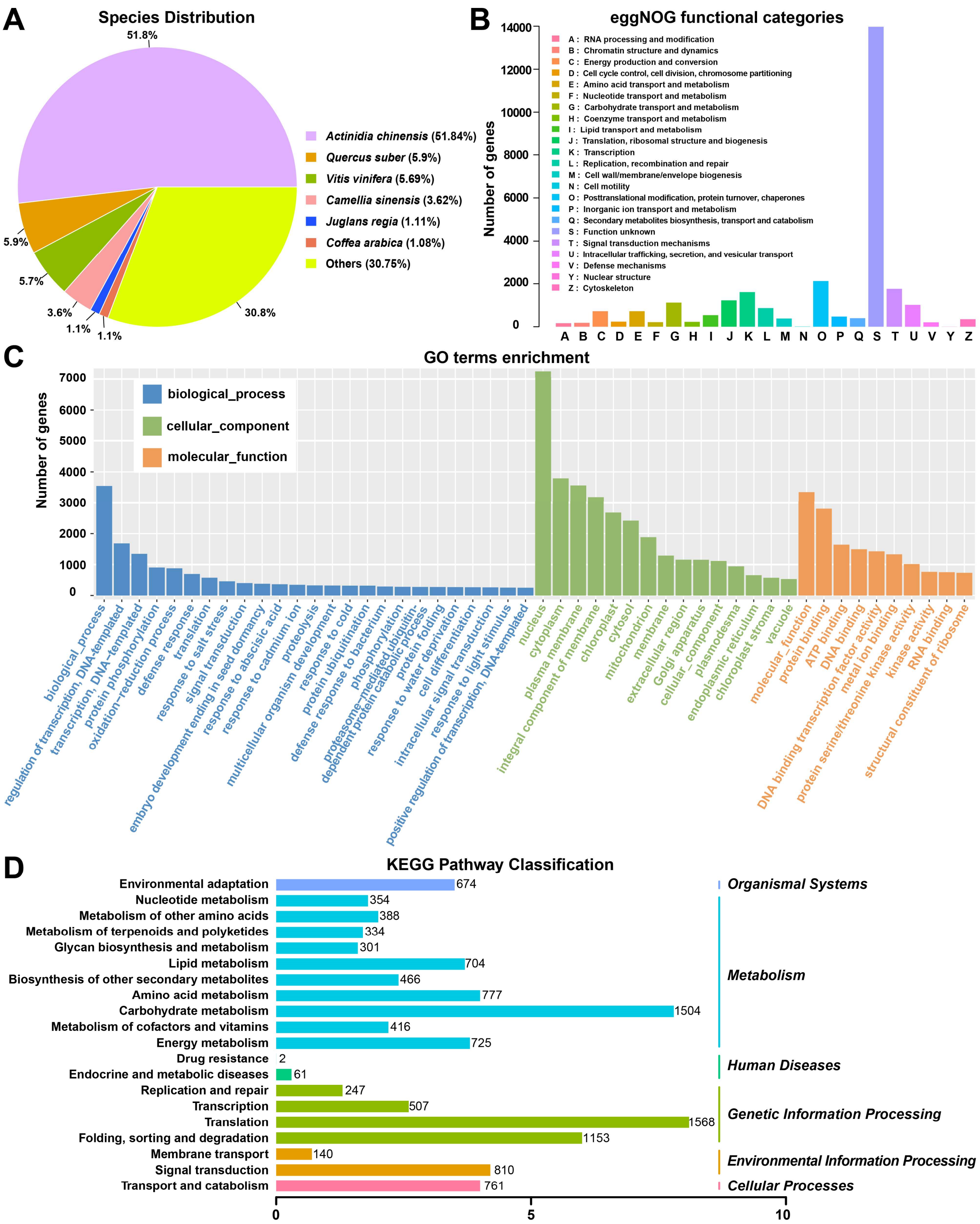

Supplementary Fig.S1 The evaluation of the functional annotation of the unigenes. (A) The distribution of unigenes annotated in the NR database. (B) The distribution of unigenes annotated in the eggNOG database. (C) The distribution of unigenes annotated in the GO database. (D) The distribution of unigenes annotated in the KEGG pathways database.
