# Supplementary material for: The developmental transcriptome dynamics of current-year shoot utilized as scion in Camellia chekiangoleosa
Source: BMC Plant Biol. 2025 May 28;25:712. doi: 10.1186/s12870-025-06715-3 (PMC12117948; doi:10.1186/s12870-025-06715-3)
Supplement: Supplementary file 2 — Supplementary Material 2 [file 12870_2025_6715_MOESM2_ESM.pdf]

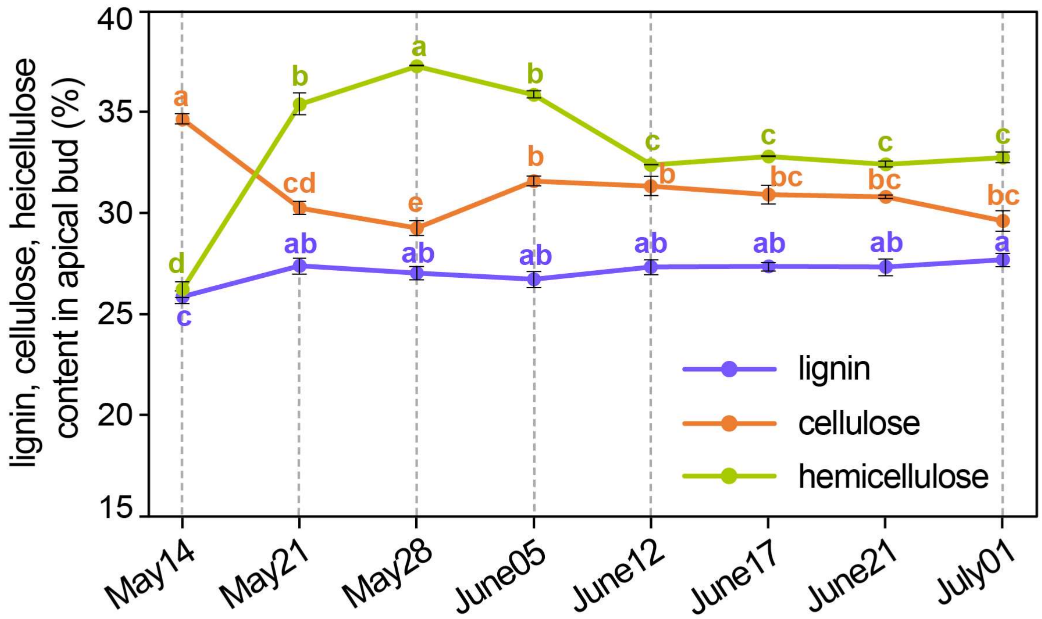

Supplementary Fig.S2 Content detection of lignin, cellulose, and hemicellulose in apical bud of *C. chekiangoleosa* current-year-shoot. Data were presented as weight percentage of the dry mass of the sample (w/w %). The presence of extra components including slight amounts of pectin and cell wall proteins, cannot be excluded. Error bars represent the mean ± SD (n = 3). Different letters indicated significant differences ($P < 0.05$) using one-way ANOVA followed by a Duncan's test.
