# Supplementary material for: The developmental transcriptome dynamics of current-year shoot utilized as scion in Camellia chekiangoleosa
Source: BMC Plant Biol. 2025 May 28;25:712. doi: 10.1186/s12870-025-06715-3 (PMC12117948; doi:10.1186/s12870-025-06715-3)
Supplement: Supplementary file 3 — Supplementary Material 3 [file 12870_2025_6715_MOESM3_ESM.pdf]

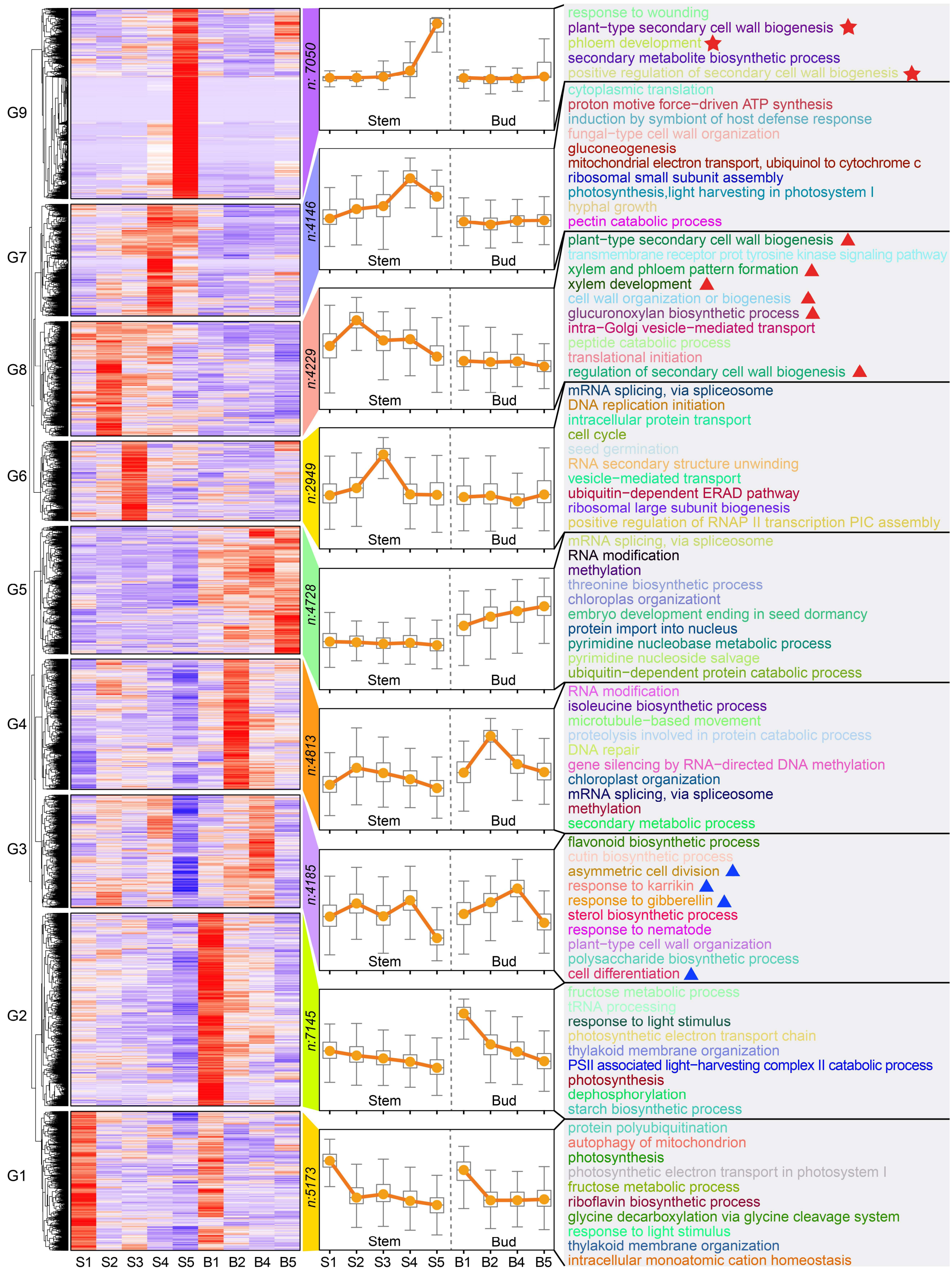

Supplementary Fig.S3 Differential expression pattern and function classification of genes among different developmental stages between stem and bud tissues during *C. chekiangoleosa* shoot development. Heatmap visualized the k-means clustering groups based on the gene expression profile. Number in vertical italicized font indicated the gene number of each cluster. Line chart showed the temporal changes of gene expression. The top 10 representative GO terms enriched significantly in genes of each cluster that output by online tool REVIGO (http://revigo.irb.hr/) were listed in the right-most.
