# Supplementary material for: The developmental transcriptome dynamics of current-year shoot utilized as scion in Camellia chekiangoleosa
Source: BMC Plant Biol. 2025 May 28;25:712. doi: 10.1186/s12870-025-06715-3 (PMC12117948; doi:10.1186/s12870-025-06715-3)
Supplement: Supplementary file 5 — Supplementary Material 5 [file 12870_2025_6715_MOESM5_ESM.pdf]

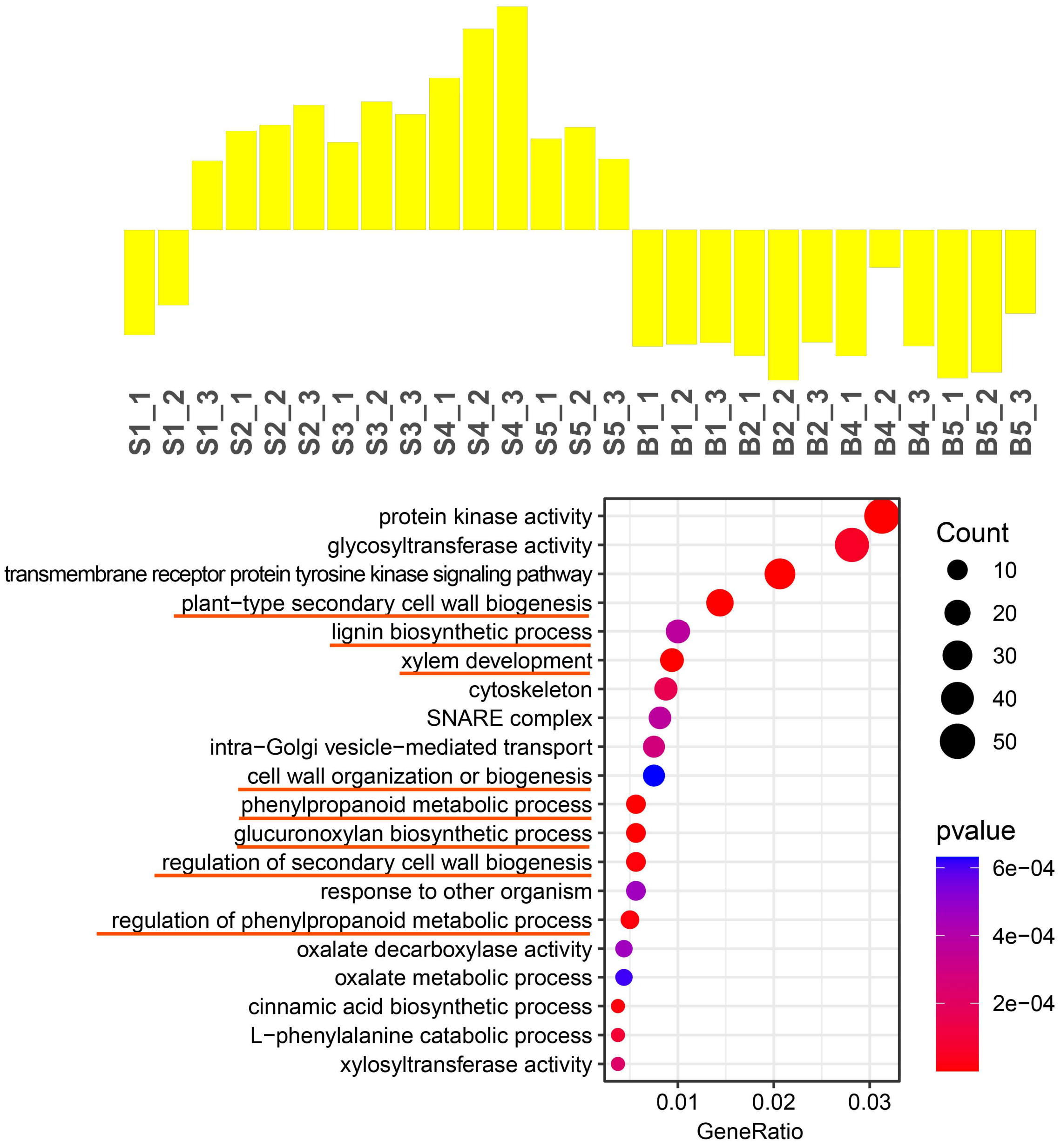

Supplementary Fig.S5 Expression profile and GO enrichment analysis of genes co-expressed in yellow module.
