# Supplementary material for: The developmental transcriptome dynamics of current-year shoot utilized as scion in Camellia chekiangoleosa
Source: BMC Plant Biol. 2025 May 28;25:712. doi: 10.1186/s12870-025-06715-3 (PMC12117948; doi:10.1186/s12870-025-06715-3)
Supplement: Supplementary file 6 — Supplementary Material 6 [file 12870_2025_6715_MOESM6_ESM.pdf]

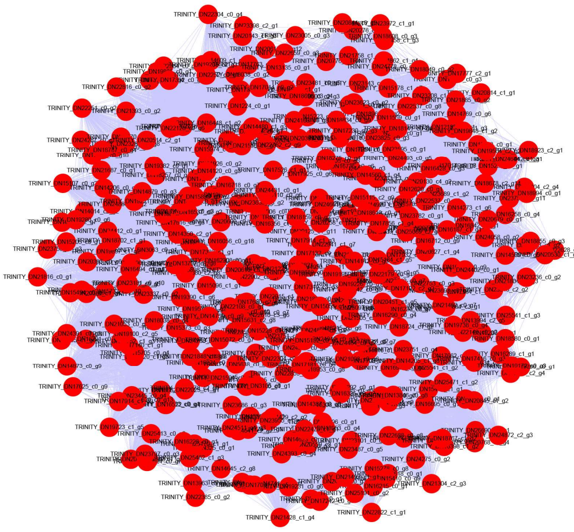

**Sub-cluster 1（node: 399, Score: 293.839）**

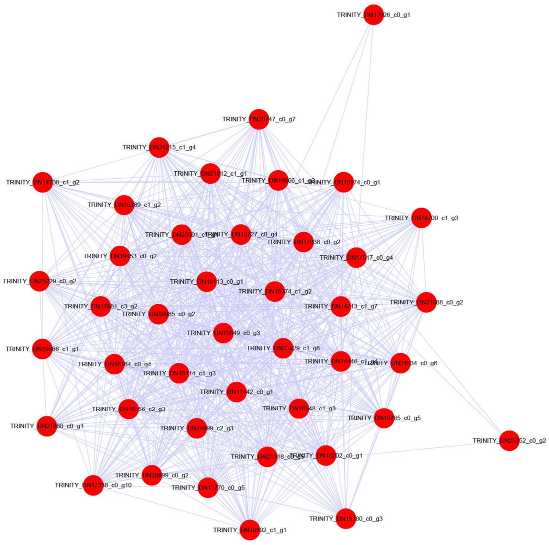

**Sub-cluster 2（node: 42, Score: 35.024）**

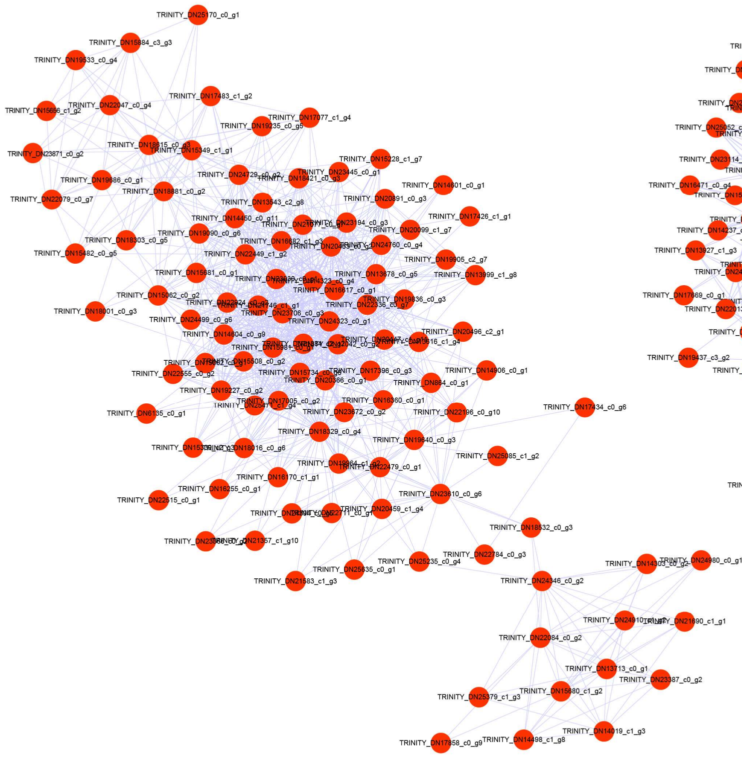

**Sub-cluster 3（node: 106, Score: 17.429）**

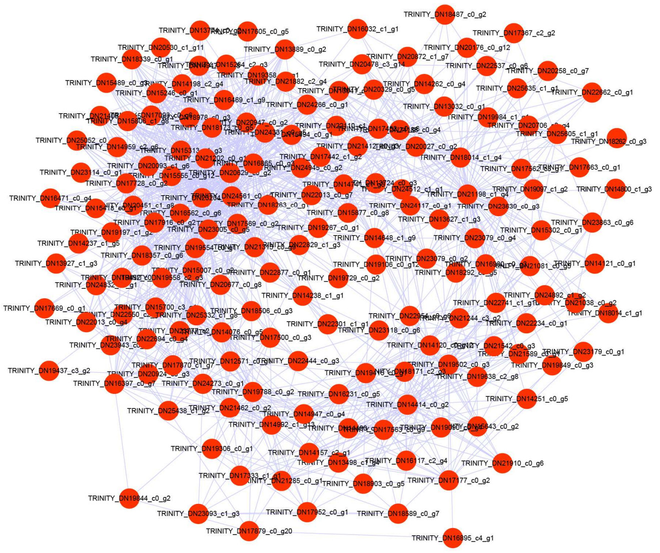

**Sub-cluster 2（node: 170, Score: 15.657）**

**Supplementary Fig.S6 The top 4 ranked sub-clusters screened from green module by MOCDE method.** Gene numbers and score of each cluster were indicated in parentheses.
