# Supplementary material for: The developmental transcriptome dynamics of current-year shoot utilized as scion in Camellia chekiangoleosa
Source: BMC Plant Biol. 2025 May 28;25:712. doi: 10.1186/s12870-025-06715-3 (PMC12117948; doi:10.1186/s12870-025-06715-3)
Supplement: Supplementary file 7 — Supplementary Material 7 [file 12870_2025_6715_MOESM7_ESM.pdf]

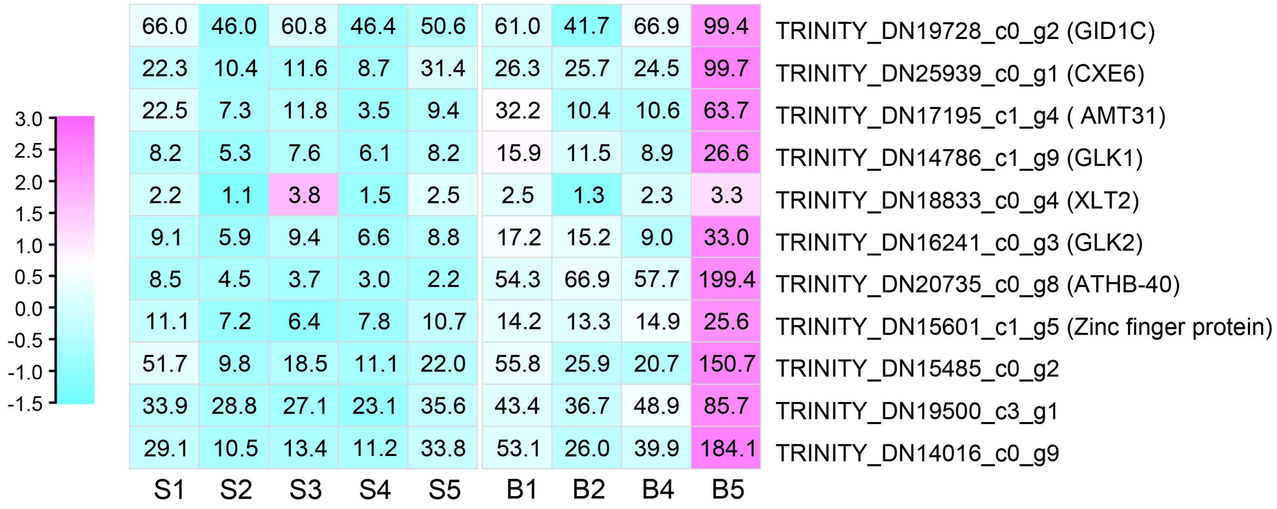

Supplementary Fig.S7 Heatmap showing the transcript abundance of the top11 hub genes in B5-related tan module.
