# Supplementary material for: The developmental transcriptome dynamics of current-year shoot utilized as scion in Camellia chekiangoleosa
Source: BMC Plant Biol. 2025 May 28;25:712. doi: 10.1186/s12870-025-06715-3 (PMC12117948; doi:10.1186/s12870-025-06715-3)
Supplement: Supplementary file 8 — Supplementary Material 8 [file 12870_2025_6715_MOESM8_ESM.pdf]

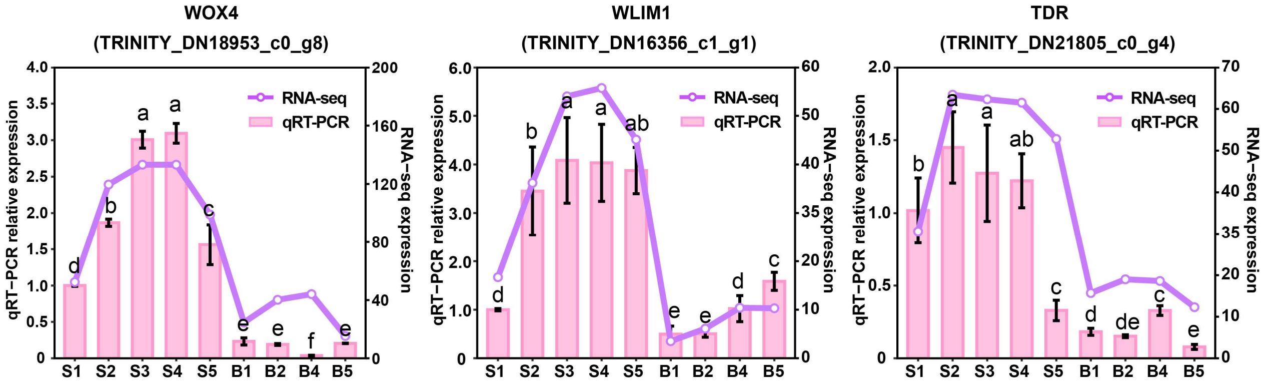

Supplementary Fig.S8 Temporal changes in transcriptional levels for candidate hub genes related to vascular development in *C. chekiangoleosa* shoot. Relative expression levels of qRT-PCR calculated using GADPH as the reference gene are shown in the right y-axis. RNA-Seq expression of the transcript (TPM) is shown in the left y-axis. Data are represented as mean ± SD (n = 3), and different letters above the columns indicated significant differences ($P < 0.05$) using one-way ANOVA followed by a Duncan's test.
